# Comparison of treatment planning approaches for spatially fractionated irradiation of deep tumors

**DOI:** 10.1002/acm2.12617

**Published:** 2019-05-21

**Authors:** Khadija Sheikh, William T. Hrinivich, Leslie A. Bell, Joseph A. Moore, Wolfram Laub, Akila N. Viswanathan, Yulong Yan, Todd R. McNutt, Jeffrey Meyer

**Affiliations:** ^1^ Department of Radiation Oncology and Molecular Radiation Sciences Johns Hopkins University School of Medicine Baltimore MD USA; ^2^ Department of Radiation Oncology University of Texas Southwestern Medical Center Dallas TX USA

**Keywords:** deep‐seated tumors, grid therapy, spatially fractionated radiotherapy

## Abstract

**Purpose:**

The purpose of this work was to compare the dosimetry and delivery times of 3D‐conformal (3DCRT)‐, volumetric modulated arc therapy (VMAT)‐, and tomotherapy‐based approaches for spatially fractionated radiation therapy for deep tumor targets.

**Methods:**

Two virtual GRID phantoms were created consisting of 7 “target” cylinders (1‐cm diameter) aligned longitudinally along the tumor in a honey‐comb pattern, mimicking a conventional GRID block, with 2‐cm center‐to‐center spacing (GRID_2 cm_) and 3‐cm center‐to‐center spacing (GRID_3 cm_), all contained within a larger cylinder (8 and 10 cm in diameter for the GRID_2 cm_ and GRID_3 cm_, respectively). In a single patient, a GRID_3 cm_ structure was created within the gross tumor volume (GTV). Tomotherapy, VMAT (6 MV + 6 MV‐flattening‐filter‐free) and multi‐leaf collimator segment 3DCRT (6 MV) plans were created using commercially available software. Two tomotherapy plans were created with field widths (TOMO_2.5 cm_) 2.5 cm and (TOMO_5 cm_) 5 cm. Prescriptions for all plans were set to deliver a mean dose of 15 Gy to the GRID targets in one fraction. The mean dose to the GRID target and the heterogeneity of the dose distribution (peak‐to‐valley and peak‐to‐edge dose ratios) inside the GRID target were obtained. The volume of normal tissue receiving 7.5 Gy was determined.

**Results:**

The peak‐to‐valley ratios for GRID_2 cm_/GRID_3 cm_/Patient were 2.1/2.3/2.8, 1.7/1.5/2.8, 1.7/1.9/2.4, and 1.8/2.0/2.8 for the 3DCRT, VMAT, TOMO_5 cm_, and TOMO_2.5 cm_ plans, respectively. The peak‐to‐edge ratios for GRID_2 cm_/GRID_3 cm_/Patient were 2.8/3.2/5.4, 2.1/1.8/5.4, 2.0/2.2/3.9, 2.1/2.7/5.2 and for the 3DCRT, VMAT, TOMO_5 cm_, and TOMO_2.5 cm_ plans, respectively. The volume of normal tissue receiving 7.5 Gy was lowest in the TOMO_2.5 cm_ plan (GRID_2 cm_/GRID_3 cm_/Patient = 54 cm^3^/19 cm^3^/10 cm^3^). The VMAT plans had the lowest delivery times (GRID_2 cm_/GRID_3 cm_/Patient = 17 min/8 min/9 min).

**Conclusion:**

Our results present, for the first time, preliminary evidence comparing IMRT‐GRID approaches which result in high‐dose “islands” within a target, mimicking what is achieved with a conventional GRID block but without high‐dose “tail” regions outside of the target. These approaches differ modestly in their ability to achieve high peak‐to‐edge ratios and also differ in delivery times.

## 1 INTRODUCTION

Spatially fractionated radiotherapy was initially used in the era of low energy x‐rays to allow for safe delivery of radiation to internal tumors while allowing for skin and superficial tissue sparing.^1^, ^2^ The radiation beam, often delivered through a single field, was spatially fractionated into small beamlets by sieve‐like blocking to form a grid pattern (GRID therapy). Tissues, such as skin, in the blocked portion of the treatment field were thought to promote healing/repair of normal tissues irradiated to high dose in the beamlet paths. In the era of skin‐sparing megavoltage photon irradiation, GRID therapy has continued to play a role in radiation oncology, mostly in the treatment of bulky tumors.^1^, ^2^ The GRID treatment has typically been delivered in one high‐dose (15–20 Gy) fraction, often followed by conventionally fractionated treatment courses which target the entire tumor. The radiation field is partitioned by commercially available blocks or by MLC leaf patterns which reproduce the effect of these blocks.^3^ The treatments are often delivered in a single field. Many studies have shown excellent tumor response results with this approach and there is a great deal of interest in the radiobiology of GRID treatments.^1^, ^2^, ^3^, ^4^, ^5^ Upfront treatment with GRID may influence oxygenation in tumors as well as induce bystander effects.^4^, ^5^

Despite the successes with conventional GRID therapy, it has dosimetric limitations in the treatment of very deep‐seated tumors. Due to attenuation characteristics, the maximum dose from a single photon beam is delivered to a shallow depth, with decreasing dose as the deep tumor is reached. Some investigators have tried to mitigate this dosimetric problem with the use of parallel opposed fields.^6^ Another solution, which maintains the unique geometry of high‐dose “islands” within tumors inherent with GRID and takes advantage of high‐energy x‐ray attenuation features, is to paint three‐dimensional target structures throughout the tumor which mimic conventional two‐dimensional GRID blocks and then use conformal or intensity‐modulated planning with the goal to deliver high doses to these areas (instead of the entire tumor). This approach has previously been studied using tomotherapy.^7^ In this report, we compare a tomotherapy‐based approach with two other approaches: volumetric modulated arc therapy (VMAT) and a simple 3D conformal (3DCRT)‐based planning technique using cylindrical target structures.

## 2 MATERIALS AND METHODS

### 2.1 GRID structures

**Figure 1:**
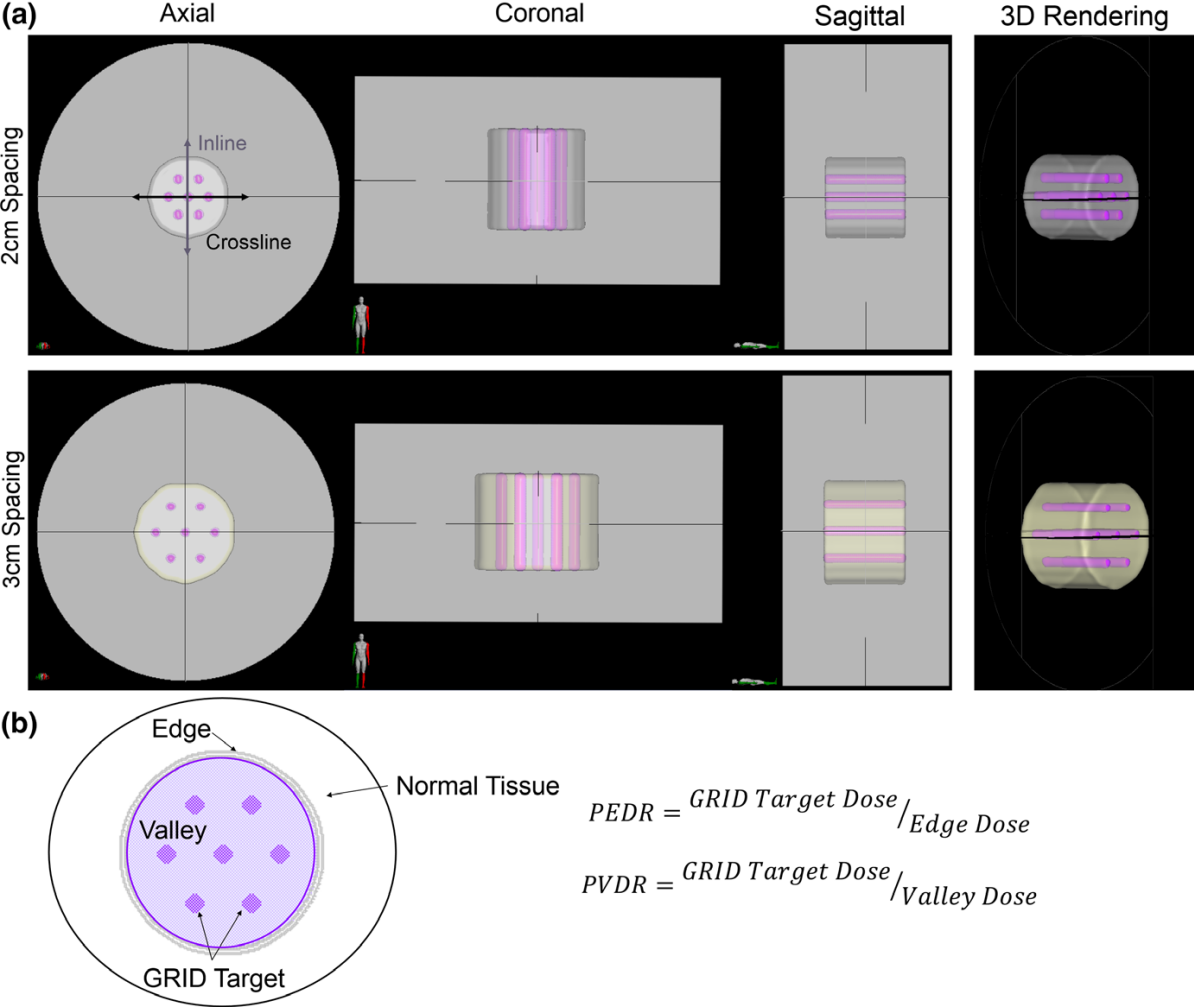
(a) Target arrangement for two virtual GRID phantoms consisting of seven cylinders (1‐cm diameter) aligned longitudinally along the GTV in a honey‐comb pattern, mimicking a conventional GRID block with 2‐cm center‐to‐center spacing (GRID_2 cm_) and 3‐cm center‐to‐center spacing (GRID_3 cm_), all contained within a larger cylinder. The larger cylinder is 8 and 10 cm in diameter for the GRID_2 cm_ and GRID_3 cm _arrangements, respectively. Inline and crossline profiles are identified by the arrows. (b) Schematic of quantities evaluated for plan assessment. GTV is defined by the purple outline, valley (which is the GTV minus the GRID target) is defined by the light purple, and the GRID target is defined by the purple filled circles within the GTV. The ring is the 2‐mm ring around the GTV. The peak‐to‐edge dose ratio (PEDR) is defined as the ratio of the mean dose to the GRID target to the mean dose of the valley. The peak‐to‐valley dose ratio (PVDR) is defined as the ratio of the mean dose to the GRID target to the mean dose to the 2‐mm edge. The solid black line is the volume of the normal tissue.

The virtual GRID structures were generated by DICOMan which is an open source software (University of Arkansas, Little Rock, Arkansas).^8^ DICOMan allows for a GRID target to be created within the target volume. The diameter of the cylinders and the center‐to‐center distance between the cylinders can be configured to the patient's anatomy. To mimic an ideal geometry, two virtual GRID phantoms were created consisting of seven cylinders (1‐cm diameter) aligned longitudinally within a larger cylinder (the “GTV”) in a honey‐comb pattern, mimicking a conventional GRID block, with 2 cm (GRID_2 cm_) and 3 cm (GRID_3 cm_) center‐to‐center spacing [Fig. 1(a)]. The larger cylinders (GTV) were 8 and 10 cm in diameter for the GRID_2 cm_ and GRID_3 cm_ arrangements, respectively. The “normal tissue” outside of the GTV was defined as a 5‐cm ring around the GTV. In the dosimetric analysis, we defined the “peak” dose as the mean dose of the GRID target. We defined the “edge” as a 2‐mm ring just outside of the GTV, and the edge dose as the mean dose to this structure.

### 2.2 Phantom treatment planning

All treatment plans were generated using the Pinnacle v. 9.10 treatment planning system (TPS) (Philips Amsterdam DE) or the tomotherapy TPS (Accuray, Sunnyvale, California). All 3DCRT, VMAT, and GRID‐block plans were planned to be delivered by an Elekta linac equipped with Agility MLC (Versa HD, Elekta Inc., Stockholm, Sweden). Three treatment techniques were used for comparison: 3DCRT, VMAT, and tomotherapy. For the GRID_2 cm_ arrangement, the 3DCRT plans used six beams with gantry angles ranging from 30 to 330° (at 60° increments) intended to align beam paths with groups of individual cylinders. For each gantry angle, three equally weighted segments were created where the field shape corresponded to the outline of a single cylinder. For the GRID_3 cm_ arrangement, the 3DCRT plan used the same gantry angles as the GRID_2 cm_ plan, with six additional beams at gantry angles 0°, 60°, 120°, 180°, 240°, and 300°. For these additional gantry angles, a single segment with the field shape corresponding to the central cylinder, with lower weight, was used.

The VMAT plans for GRID_2 cm_ used four full arcs with collimator rotated at 90° (for arcs 180°–182°) and 270° (for arcs 182°–180). These collimator angles were selected to minimize the creation of “dose islands” between grid targets at varying gantry angles. The VMAT GRID_3 cm _arrangement used two full arcs with the collimator rotated at 90° (for the 180°–182° arc) and 270° (for the 182°–180 arc). For tomotherapy, two plans were created with field widths of 5.01 cm (TOMO_5 cm_) and 2.5 cm (TOMO_2.5 cm_) with the same pitch of 0.43. Table 1 shows a list of target and planning structure objectives used for the VMAT and tomotherapy plans. We used 6 MV energy for all plans.

**Table 1:** List of target and planning structures for VMAT and Tomotherapy plans.

Structures	Objectives	Weights
GRID_2 cm _VMAT		
Target	D_min_ = 15 Gy	50
Valley	D_max_ = 2 Gy	0.01
Rings	D_max_ = 12 Gy	5
GRID_2 cm_ Tomotherapy		
Target	D_min_ = 15 Gy	1000
Valley	D_max_ = 9.5 Gy	100
Valley	D_10_ = 9 Gy	100
Valley	D_20_ = 8 Gy	100
Rings	D_max_ = 12 Gy	200
GRID_3 cm _VMAT		
Target	D_min_ = 15 Gy	50
Valley	D_max_ = 2 Gy	0.001
Rings	D_max_ = 8 Gy	0.008
GRID_3 cm_ Tomotherapy		
Target	D_min_ = 15 Gy	1000
Valley	D_max_ = 8.5 Gy	50
Valley	D_10_ = 8 Gy	50
Valley	D_20_ = 7 Gy	50
Rings	D_max_ = 12 Gy	100
Patient VMAT and Tomotherapy		
Target	Dmin = 15 Gy	1/1000
Valley	Dmax = 9.5 Gy	0.3/50
Valley	D10 = 9 Gy	0.3/50
Valley	D20 = 8 Gy	0.3/50
Rings	Dmax = 10 Gy	0.05/100
Large ring	Dmax = 3 Gy	0.05/150

D_min_, minimum dose; D_max_, maximum dose; D_10_, dose to 10% of the volume; D_20_, dose to 20% of the volume, weights of the patient plan are expressed as VMAT/Tomotherapy.

### 2.3 Patient treatment planning

For a single patient with recurrent cervical cancer, a waiver of consent was obtained through the Johns Hopkins Hospital institutional review board. Images were acquired using a Brilliance Big Bore CT simulator (Philips, Amsterdam DE) and transferred to the Pinnacle TPS for contouring and planning. Patient GTV and organs‐at‐risk were contoured by the attending physician.

The same patient CT image datasets and same contours generated from Pinnacle TPS were transferred to DICOMan to generate a virtual GRID_3 cm_ arrangement.

The prescription for the GRID_block _was at a point at the depth of maximum dose (d_max_) along the central axis. Five treatment plans were created for comparison: 3DCRT with and without optimized segment weighting (6 MV), VMAT (6 MV with and without FFF), and tomotherapy. The 3DCRT plans used six beams with the following gantry angles: 20°, 70°, 90°, 110°, 270°, and 340°, again selected to align the beam path with groups of grid targets. Each beam had three to five segments where each segment had a field shape associated with a cylinder. The weighted 3DCRT plan had lower weights to the posterior beams to lower dose to the cauda equina. The VMAT plans for both the GRID_2 cm_ and GRID_3 cm_ arrangements used two full arcs with the collimator rotated at 90°. Three millimeter wide rings were created around the GRID structures. Surrounding the PTV, a 5‐cm wide ring was created to limit dose to the normal tissue. For tomotherapy, two plans were created with field widths of 5.01 cm (TOMO_5 cm_) and 2.5 cm (TOMO_2.5 cm_) with the same pitch of 0.43. Table 1 shows the objectives for the VMAT and tomotherapy plans.

To illustrate the difference between the IMRT and 3DCRT GRID approaches and a conventional single field GRID‐block approach, a conventional GRID‐block treatment plan was also produced for this patient. We used an in‐house compensator model for a commercially available GRID block (.decimal inc., Sanford FL) commissioned for use in our clinic. The compensator models the geometry, material, and thickness of the brass GRID block, enabling the analysis of resultant 3D dose distributions in Pinnacle plan was produced using a single angle. Prescribed dose for phantom and patient was set to a mean 15 Gy to the GRID target in a single fraction. Patient plans were delivered on a MapCHECK phantom for patient‐specific quality assurance. Using the γ index tolerance criteria of 3%/3 mm, all plans passed at a threshold of ≥95%.

### 2.4 Dosimetry analysis

The peak‐to‐valley dose ratio (PVDR) was defined as the ratio of the mean dose to the GRID target to the mean dose of the valley. The peak‐to‐edge dose ratio (PEDR) was defined as the ratio of the mean dose to the GRID target to the mean dose to the 2‐mm ring [Fig. 1(b)]. The volume of the normal tissue receiving 7.5 Gy (V_7.5 Gy_) and 5 Gy (V_5 Gy_) was quantified. To evaluate superficial tissue sparing, we evaluated the dosimetric differences for 0.03 cc (D_0.03 cc_) and 10 cc (D_10 cc_) of skin for the patient data.

## 3 RESULTS

**Figure 2:**
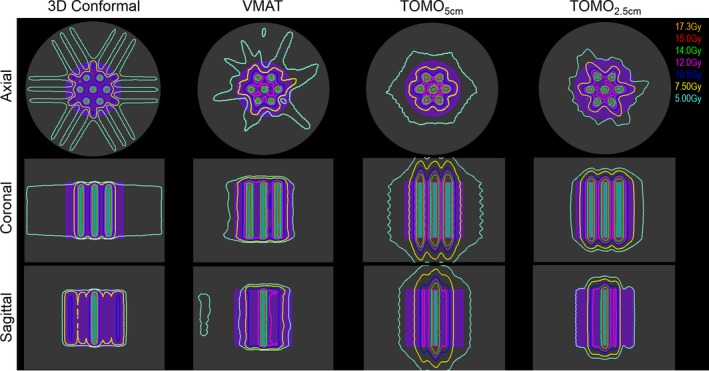
3D conformal (3DCRT), VMAT, and Tomotherapy (with field width 5 cm [TOMO_5 cm_] and 2.5 cm [TOMO_2.5 cm_]), plans forming the grid pattern using GRID_2 cm_. The GRID_2 cm_ target is indicated by the cyan and the GTV is shown in purple color wash. The distance from the GRID_2 cm_ target to the edge of the GTV is 2 cm. Mean dose of 15 Gy was prescribed to the GRID_2 cm _target.

Figure 2 shows a comparison of the 3DCRT, VMAT, and tomotherapy plans across the GRID_2 cm_ arrangement. The coronal, axial, and sagittal planes are shown. In the axial slice, it is visually apparent that the dose to edge of the GTV is highest in the 3DCRT plan in the left‐right and anterior‐posterior directions. However, the dose outside the GTV in the superior‐inferior direction and the dose to the valley appear to be the lowest in the 3DCRT plan. In the coronal and sagittal planes, it appears that the TOMO_5 cm_ has the highest dose outside the GTV in the superior‐inferior and left‐right directions. Quantitatively, the 3DCRT plan had the lowest normal tissue V_7.5 Gy _and TOMO_2.5 cm _plan had the lowest normal tissue V_5 Gy _for the GRID_2 cm_ arrangement (Table 2). Compared to the 3DCRT and VMAT plans, both TOMO_5 cm_ and TOMO_2.5 cm _plans had the longer delivery times.

**Table 2:** Comparison of delivery time, peak‐to‐edge dose and peak‐to‐valley dose ratios for 3D conformal, VMAT, and Tomotherapy plans across the GRID_2 cm_ and GRID_3 cm_ cylinder arrangements.

	3D CRT	VMAT	TOMO_5 cm_	TOMO_2.5 cm_
2‐cm separation				
Delivery time (min)	15	17	37	56
GRID D_mean_ (Gy)	15	15	15	15
GTV edge D_mean_ (Gy)	5.3	7.3	7.6	7.1
GTV valley D_mean_ (Gy)	7.2	8.7	8.8	8.4
PEDR	2.8	2.1	2.0	2.1
PVDR	2.1	1.7	1.7	1.8
Normal tissue V_7.5 Gy _(cc)	3	30	110	54
Normal tissue V_5 Gy _(cc)	334	363	461	222
3 cm separation				
Delivery time (min)	21	8	24	45
GRID D_mean_ (Gy)	15	15	15	15
GTV edge D_mean_ (Gy)	4.7	8.3	6.7	5.5
GTV valley D_mean_ (Gy)	6.5	10	7.9	7.6
PEDR	3.2	1.8	2.2	2.7
PVDR	2.3	1.5	1.9	2.0
Normal tissue V_7.5 Gy _(cc)	30	108	43	19
Normal tissue V_5 Gy _(cc)	291	647	767	178

3DCRT, three‐dimensional conformal radiotherapy; TOMO_5 cm_, tomotherapy plans with 5 cm field width; TOMO_2.5 cm_, tomotherapy plans with 2.5 cm field width; D_mean_, mean dose of structure; D_max_, maximum dose to structure; PEDR, peak‐to‐edge dose ratio; PVDR, peak‐to‐valley dose ratio; Normal Tissue V_7.5_, volume of normal tissue receiving 7.5 Gy; Normal Tissue V_5_, volume of normal tissue receiving 5 Gy.

**Figure 3:**
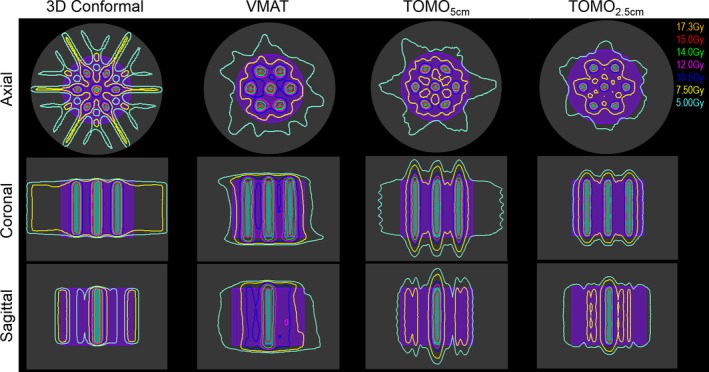
3DCRT, VMAT, and Tomotherapy (with field width 5 cm [TOMO_5 cm_] and 2.5 cm [TOMO_2.5 cm_]), plans forming the grid pattern using GRID_3 cm_. The GRID_3 cm_ target is indicated by the cyan and the GTV is shown in purple color wash. The distance from the GRID_3 cm_ target to the edge of the GTV is 2 cm in the anterior‐posterior and superior‐inferior direction. Mean dose of 15 Gy was prescribed to the GRID_3 cm _target.

The treatment plans for the GRID_3 cm_ cylinder arrangement are shown in Fig. 3. Similar to the GRID_2 cm_ arrangement, it is visually apparent that the dose to edge of the GTV is highest in the 3D conformal plan in the left‐right and anterior‐posterior directions. Given the greater spacing between the GRID targets, the points at which the beams intersected coincide with the edge of the GTV. In the coronal and sagittal planes, it again appears that the TOMO_5 cm_ has the highest dose outside the GTV in the superior‐inferior and left‐right directions. For the GRID_3 cm_ arrangement, TOMO_2.5 cm _had the lowest normal tissue volumes receiving 7.5 and 5 Gy (Table 2). Similar to the plans for the GRID_2 cm_ arrangement, the TOMO_5 cm_ and TOMO_2.5 cm_ plans had the longest delivery times.

**Figure 4:**
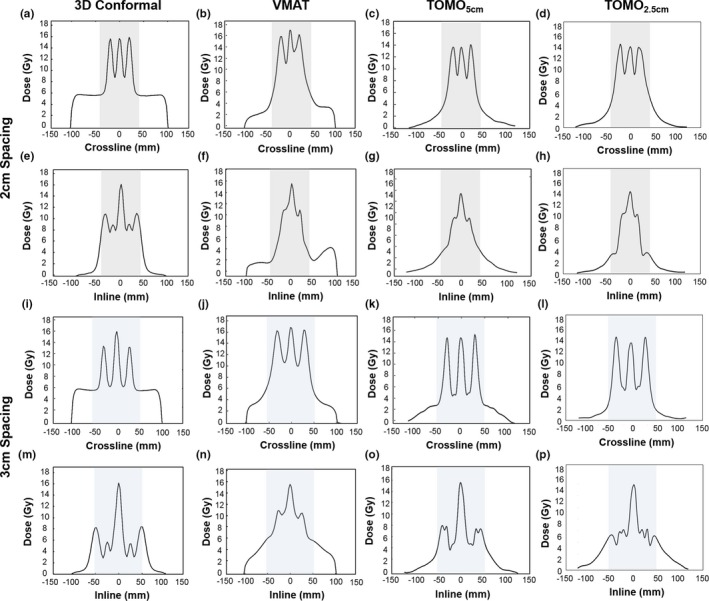
Crossline and inline profiles for 3D conformal, VMAT, and Tomotherapy (with field width 5 cm [TOMO_5 cm_] and 2.5 cm [TOMO_2.5 cm_]) for both GRID_2 cm_ and GRID_3 cm_. For the GRID_2 cm_ arrangement, crossline profiles are shown from (a–d) and inline profiles are shown from (e–h). For the GRID_3 cm_ arrangement, crossline profiles are shown from (i–l) and inline profiles are shown from (m–p). The shaded area identifies the region within the GTV.

Figure 4 shows the inline and crossline profiles for both GRID_2 cm_ and GRID_3 cm_ cylinder arrangements along the central axis. The shaded region identifies the limits of the GTV. The profiles affiliated with the GRID_3 cm_ arrangement had the lowest doses to the valleys. The mean delivery times were consistently lower for the GRID_3 cm_ arrangement for all plans (Table 2). The 3DCRT had the greatest PEDR and PVDR values, relative to the VMAT and TOMO_5 cm_, and TOMO_2.5 cm_ plans. However, the TOMO_2.5 cm_ plan had the lowest fraction of normal tissue receiving 7.5 Gy for both the GRID_2 cm_ and GRID_3 cm_ arrangements.

**Figure 5:**
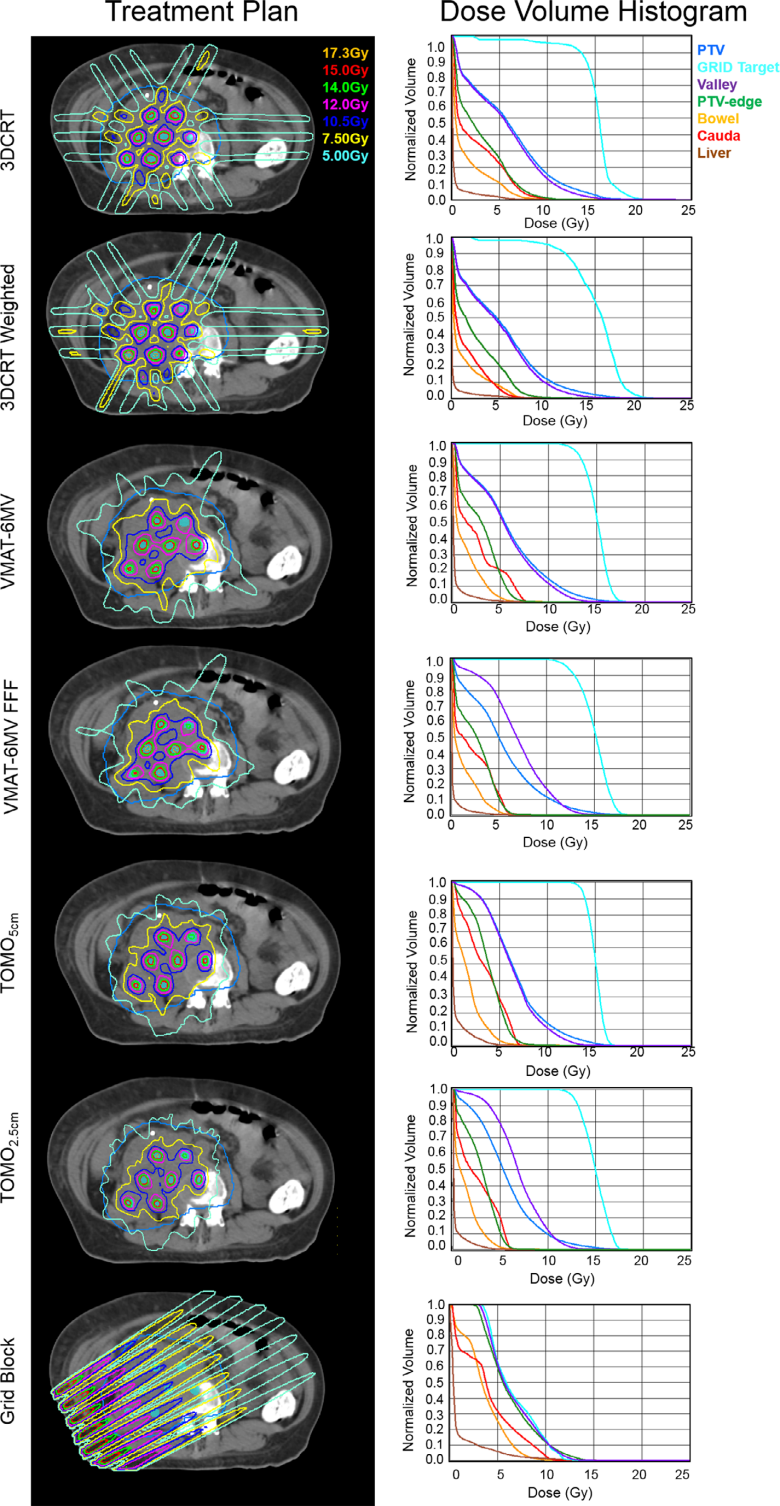
3DCRT, VMAT, Tomotherapy, and GRID‐block plans across the GRID_3 cm_ cylinder arrangement and corresponding dose volume histograms for a representative subject. Axial slices are shown where the teal contour represents the PTV and the cyan represents the GRID_3 cm_.

For a representative patient, a single axial slice is shown for the six treatment plans in Fig. 5. Visually, it can be seen that the VMAT and tomotherapy plans resulted in the lowest dose surrounding the PTV. Similar to the phantom plans, the 3DCRT plans had the highest PEDR and PVDR (Table 2 and Table 3). However, when looking at the percentage of normal tissue receiving 7.5 and 5.0 Gy, these values were lowest with the VMAT and tomotherapy plans (Table 3). Specifically, the TOMO_2.5 cm_ plan had the lowest D_max_ to the cauda (6.8 Gy) and liver (5.4 Gy), whereas the 3DCRT plan had the highest (cauda D_max_ = 13.4 Gy and liver D_max_ = 10.3 Gy). As expected, the 3DCRT plans had the highest dose to the skin (D_0.03cc_ > 9 Gy and D_10cc_ = 5.6 Gy). We created a VMAT plan using the 10MV‐FFF; however, it was not deliverable due to the dose rate variability. We were able to deliver a 6 MV‐FFF plan. Out of the deliverable plans, the VMAT plan using 6FFF energy had the lowest dose to skin (D_0.03cc_ = 4.5 Gy and D_10cc_ = 3.1 Gy) and bowel (D_max_ = 8.8 Gy). It should be noted that the TOMO_2.5 cm_ field width had the highest delivery time (34min). The overall lowest doses to the critical structures were observed in the VMAT‐6FFF plan, which also had the lowest delivery time (5 min). Figure 5 also shows the treatment plan utilizing the GRID_block_. As expected, the dose to the deep‐seated targets is visually lower than that of the other treatment plans. The dose to the normal tissues outside the GTV is visually higher compared to the other plans.

**Table 3:** For a representative subject: comparison of delivery time, peak‐to‐edge dose and peak‐to‐valley dose ratios, and organs at risk for 3D conformal, VMAT, and Tomotherapy plans across the GRID_3cm_ cylinder arrangement

	3DCRT	3DCRT WT	VMAT 6MV	VMAT 6FFF	VMAT 10FFF	TOMO_5cm_	TOMO_2.5cm_
Delivery Time (min)	16	17	9	5	N/A	19	34
Target volumes							
GRID D_mean_ (Gy)	15	15	15	15	15	15	15
GRID D_max_ (Gy)	20.1	21.1	17.9	18.4	18.5	17	18.4
GRID D_95_ (Gy)	11.2	10.2	12.9	12.2	12.6	13.6	12.7
GTV D_mean_ (Gy)	5.6	5.1	5.8	5.4	5.4	6.5	5.6
GTV Edge D_mean_ (Gy)	2.8	2.5	2.9	2.6	2.5	3.8	2.9
GTV Edge D_max_(Gy)	13.6	12.6	8.6	7.7	7.6	10.9	8.7
GTV Valley D_mean_ (Gy)	5.3	4.7	5.5	5.0	5.0	6.3	5.3
GTV Valley D_max_ (Gy)	23.4	18.6	15.8	15.7	16.3	15.7	17.1
PEDR	5.4	6.0	5.4	5.8	6.0	3.9	5.2
PVDR	2.8	3.1	2.8	3.0	3.0	2.4	2.8
Normal tissue							
Liver D_max_ (Gy)	9.8	10.3	7.5	5.6	5.5	6.5	5.4
Liver D_mean_ (Gy)	0.3	0.2	0.3	0.2	0.2	0.4	0.3
Cauda D_max_ (Gy)	13.4	9.7	7.8	7.6	7.5	7.4	6.8
Cauda D_mean_ (Gy)	2.3	1.5	2.5	2.0	1.5	3.4	2.5
Bowel D_max_ (Gy)	15.7	9.8	9.4	8.8	8.9	11.3	9.1
Bowel D_mean_ (Gy)	1.4	1.2	1.3	1.2	1.2	1.6	1.3
Skin D_0.03cc_ (Gy)	9.5	9.4	4.9	4.5	4.6	5.0	4.6
Skin D_10cc_ (Gy)	5.6	5.6	3.3	3.1	2.8	4.1	3.4
Normal tissue V_7.5Gy_ (cc)	124	79	17	9	7	51	10
Normal tissue V_5Gy_ (cc)	777	625	321	194	164	479	211

3DCRT: 3‐dimensional conformal radiotherapy; 3DCRT WT: 3‐dimensional conformal radiotherapy plan with unequal weighted beams; FFF: flattening filter free; TOMO_5cm_: tomotherapy plans with 5cm field width; TOMO_2.5cm_: tomotherapy plans with 2.5cm field width; D_mean_: mean dose of structure; D_max_: maximum dose to structure; D_95_: dose to 95% of the volume; PEDR: peak‐to‐edge dose ratio; PVDR: peak‐to‐valley dose ratio; Normal Tissue V_7.5_ volume of normal tissue receiving 7.5 Gy; Normal Tissue V_5_: volume of normal tissue receiving 5 Gy.

## 4 DISCUSSION

In this study, we used tomotherapy‐, VMAT‐, and 3DCRT‐based approaches to generate spatially fractionated radiation treatment patterns in deep‐seated tumors. We painted cylindrical targets throughout tumor volumes as a template to guide treatment planning. These cylinders were a 3D representation of the 2D circular patterns seen with conventional GRID blocks. In the initial phantom studies, the simple 3DCRT approach for the GRID_2 cm_ and GRID_3 cm_ arrangements had the highest PEDR and PVDR values, relative to the results seen with the VMAT and tomotherapy plans. With the selected gantry angles and blocking technique, much of the valley could be directly blocked with the 3D planning approach.

Although the 3D conformal demonstrated high PEDR and PVDR values in the example patient plan, these metrics do not quantify the extent of dose that is delivered outside the GTV. We characterized dose spillage in normal tissues by evaluating V_7.5 Gy_, and V_5 Gy_ values. These values were the lowest in the TOMO_2.5 cm _plan for both phantom and patient plans. Overall, for the patient plan, we found that the 3DCRT was inferior to the tomotherapy and VMAT approaches for these same measures. It should be noted that segment weights were distributed uniformly in the phantom due to the radial symmetry of the phantom and target, leading to minimum low dose spread in normal tissue. Asymmetric target geometry, heterogeneous density, and asymmetric avoidance structures in the patient required variable segment weights, which led to an increase in low‐dose spread in normal tissue compared to the phantom. This difference illustrates that, in relatively homogeneous media with simple target shapes a simple 3DCRT approach may provide competitive PEDR, PVDR and dose spillage results, but in practice, more advanced IMRT techniques may be required to handle complex geometries and fluctuating densities.

For the GRID_2 cm_ and GRID_3 cm_ phantom arrangements, the PEDR achieved with tomotherapy was intermediate between the 3D conformal and VMAT approaches. In the patient example, however, the PEDR was lowest with both TOMO_5 cm_ and TOMO_2.5 cm _plans. Visually, the regions with the greatest dose to the edge of the GTV were in the cranial‐caudal direction. This is consistent with the notion that in tomotherapy, the beam width remains fixed for the duration of the treatment, thus a complete field width is irradiated at the cranial and caudal end of the target, leading to higher doses at the edge relative to the 3DCRT and VMAT plans. To mitigate this, a smaller field width (2.5 cm) was also used for planning. This improved results relative to the 5‐cm field width plans, but at the consequence of a greater delivery time. Alternatively, TomoEdge technology can be used where the superior and inferior jaw opens and closes independently at the start and end of a target in order to reduce the longitudinal penumbra.^9^ This technology is not available at our institution and was not modeled. A third approach to this problem would be to truncate the GRID target volumes at their extremes near the edge of the GTV. This would lower the PEDR, as well as lower the V_5 Gy_ and V_7.5 Gy_ normal tissue values, at the expense of lowering the volume of tumor receiving high‐dose irradiation.

A key clinical issue moving forward is to determine the appropriate number and spacing of “high‐dose islands” targets within tumors. Although the value of a high PEDR result seems clear, the relevance of high PVDR values is less clear. It should be noted that the optimal PVDR and PEDR have not been extensively explored in previous work, studies have reported valley to peak ratios ranging from 0.0008 to 2.5^7^, ^10^, ^11^ and PEDRs ranging from 5 to 20.^11^ Our approach in this work was to construct, in three‐dimensions, the two‐dimensional pattern achieved with a conventional GRID‐block based on the historical successes with this approach. However, it should also be noted that in conventional GRID irradiation that, for a given slice perpendicular to the axis of the beam, the dose homogeneity in the tumor increases with depth. Thus, at the 2D level, the PVDR approaches 1 at depth. Finally, we should also acknowledge that the treatment planning time required for a GRID‐block treatment is substantially less than approaches proposed in this study. This may limit our technique to patients who do not need to be treated immediately.

In summary, we demonstrated that all of the studied approaches are capable of delivering high‐dose radiation to cylindrical structures within large tumors, yielding spatially fractionated radiation dose distributions over the length of the tumor. Selection of one approach over another may depend on the shape and depth of the GTV in the patient and the type and extent of surrounding critical structures. To evaluate the efficacy of these approaches in patients, clinical trials are required.

## CONFLICT OF INTEREST

Authors have nothing to declare.
